# An Item-Level Analysis of the Posttraumatic Stress Disorder Checklist and the Posttraumatic Growth Inventory and Its Associations With Challenge to Core Beliefs and Rumination

**DOI:** 10.3389/fpsyg.2018.02346

**Published:** 2018-12-10

**Authors:** Catarina Ramos, Isabel Leal, Pedro Alexandre Costa, Ana Rosa Tapadinhas, Richard G. Tedeschi

**Affiliations:** ^1^William James for Research, ISPA – Instituto Universitário, Lisbon, Portugal; ^2^Hospital de São Francisco Xavier, Centro Hospitalar de Lisboa Ocidental, Lisbon, Portugal; ^3^Department of Psychology, University of North Carolina, Charlotte, NC, United States

**Keywords:** posttraumatic stress disorder, core beliefs, rumination, posttraumatic growth, inter-item analysis, breast cancer

## Abstract

**Background:** Previous studies have found that rumination and challenge to core beliefs may have a predictive effect on Posttraumatic Stress Disorder (PTSD) and Posttraumatic Growth (PTG) among different samples. In addition, there is some evidence that these variables have different effects on PTSD and PTG, although the latter construct has been the target of a larger body of research and theoretical models. The main objective of the current study is to examine the effect of challenge to core beliefs, intrusive rumination, and deliberate rumination on PTSD and PTG, through an item-level analyses.

**Methods:** The sample was composed of 205 Portuguese women who had been given a breast cancer diagnosis (*M* = 54.32, *SD* = 10.05), and who completed the following self-administered questionnaires: the Posttraumatic Stress Disorder Checklist (PCL-C); the Posttraumatic Growth Inventory (PTGI); the Core Beliefs Inventory; and the Event Related Rumination Inventory. Two multivariate multiple regression analyses, using each item of the PCL-C and the PTGI as dependent variables, were conducted.

**Results:** The results demonstrated that challenges to core beliefs predict 17 of the 21 PTGI items and 12 of the 17 PCL-C items. All but one item of the PCL-C are predicted by intrusive rumination, while the variance of only 4 items of the PTGI are explained by deliberate rumination.

**Conclusion:** These findings indicate that women with breast cancer who tend to display higher levels of intrusive rumination are more likely to report PTSD symptoms, and that an examination of one’s core beliefs is predictor of both positive and negative outcomes. In spite of the proven effect of challenge to core beliefs on both variables, this study suggests that this effect has only a minor influence on PTSD, in addition to confirming its major impact on PTG.

## Introduction

The positive changes perceived as a result of the personal struggle with a traumatic event are described as Posttraumatic Growth (PTG) (Tedeschi and Calhoun, 1996, 2004). The most comprehensive model of PTG theorizes that the foundation for the possibility of growth is based on the degree to which the person’s assumptive world is shattered by a traumatic event (Tedeschi and Calhoun, 2004). The catastrophic nature of a traumatic event fosters challenges to core beliefs and the beginning of a cognitive process concerning the traumatic experience that rebuilds one’s challenged assumptive world (Danhauer et al., 2013). The assumptive world is defined as a broad set of general cognitive schemas, which represents our understanding of ourselves, others, and our world (Janoff-Bulman, 2006). Therefore, as structural components of the assumptive world, core beliefs are defined as encompassing our fundamental assumptions about the universe, connections with others, and one’s place within them, all of which determine how people will behave and direct their efforts to influence events (Janoff-Bulman, 2006; Cann et al., 2010; Taku et al., 2015). Several studies have found a strong and direct relationship between the degree of disruption of one’s core beliefs and the emergence of PTG (Cann et al., 2010; Lindstrom et al., 2013; Su and Chen, 2015; Taku et al., 2015), in addition to discovering that challenge to one’s core beliefs was the main predictor of PTG (Triplett et al., 2012; Danhauer et al., 2013; Wilson et al., 2014; Zhou et al., 2015).

The process of rebuilding a viable assumptive world, when the previous one has been shattered or disrupted as a result of a highly stressful event, could involve cognitive-emotional processing that may bring about personal growth (Janoff-Bulman, 2006). Rumination is a key element of the cognitive processing that occurs after an examination of one’s core beliefs. Intrusive rumination about an event occurs in the aftermath of a stressful life experience, whereas deliberate rumination about the implications of an event in one’s life is likely to occur at a later time, following a traumatic event (Cann et al., 2011). Theory and research have both indicated that deliberate rumination involves cognitive personal efforts to examine the event and its repercussions, and to restore and/or to restructure core beliefs (Cann et al., 2011). During this cognitive process, people often perceive positive changes or PTG in several areas of one’s life (Taku et al., 2015). In fact, the examination of core beliefs and deliberate rumination are recognized as elements crucial to the manifestation of PTG, as confirmed by a previous item-level analysis of the Posttraumatic Growth Inventory (PTGI) items (Taku and Oshio, 2015). Deliberate rumination has been shown to be positively correlated with PTG, a finding supported by other empirical studies (Stockton et al., 2011; Dong et al., 2015; Su and Chen, 2015), in addition to being more strongly associated with PTG than intrusive rumination (Morris and Shakespeare-Finch, 2011; Lindstrom et al., 2013; Zhou et al., 2015).

Intrusive rumination, in contrast, may not directly predict PTG, but it is nevertheless an important factor to the PTG process. Intrusive rumination encourages further cognitive processing of the traumatic event (i.e., deliberate rumination), which is the antecedent of PTG (Zhou et al., 2015). However, the results found in the literature showed some disparities regarding the relationship between intrusive rumination and PTG. In some studies, intrusive rumination was not significantly associated with PTG (Triplett et al., 2012; Dong et al., 2015), while another study found the existence of a negative relationship between them (Zhang et al., 2013).

In the aftermath of a traumatic event, intrusive thinking and other negative psychological outcomes, such as PTSD symptoms, are common and are widely documented (Zhou et al., 2018). There is strong evidence of the co-occurrence of PTSD and PTG in trauma survivors (Taku et al., 2008; Dekel et al., 2012; Wu et al., 2016), suggesting that both constructs are two ends of the same continuum, which share developmental mechanisms (Frazier et al., 2001). However, inconsistencies remain regarding the existence and meaning of the relationship between the two phenomena (Salsman et al., 2009). Other empirical evidence indicates the absence of a significant association between PTG and PTSD symptoms (Cordova et al., 2001, 2007; Chan et al., 2011; Ho et al., 2011) or distress (Morris and Shakespeare-Finch, 2011; Liu et al., 2014), particularly in samples with breast cancer women.

Distress and Posttraumatic Stress Disorder (PTSD) were shown to be associated with challenge to core beliefs in several studies, which examined a variety of populations (Triplett et al., 2012; Wilson et al., 2014; Zhou et al., 2015), but not in a study using a sample of women with breast cancer. A previous study among adolescents indicated that challenge to core beliefs strongly predict both the developmental pathways of PTG and PTSD, and that intrusive rumination is only associated with the development of PTSD, while deliberate rumination is only able to directly predict PTG (Zhou et al., 2015). However, the specific mechanism underlying the developmental process of each of the constructs, and the degree of influence of each variable of the PTG model on both PTG and PTSD, has not yet been examined.

An inter-item analyses of the PTGI items and the Posttraumatic Stress Disorder Checklist (PCL-C) items, as a detailed analysis of the inter-item relationship, will allow for a more comprehensive understanding of how the influence of each of the PTG model variables (i.e., challenges to core beliefs, intrusive rumination, and deliberate rumination) is related to the development of PTG and PTSD, that may be activated by experiencing an unexpected traumatic event such as breast cancer. The main objectives of the current study are: (a) to analyze the relation between the main variables, namely PTG and PTSD among a sample of Portuguese women with breast cancer, through a multiple regression analysis; (b) to determine the degree to which each PTGI item is related to challenge to one’s core beliefs and to deliberate rumination, through a 21 inter-item analyses; (c) in addition to determining the degree to which each PCL-C item is related to challenge to one’s core beliefs and to intrusive rumination, through a 17 inter-item analyses.

## Materials and Methods

### Participants

The sample participants were exclusively comprised of Portuguese women with a non-metastatic breast cancer diagnosis. The other inclusion criteria consisted of: receiving one’s first diagnosis of breast cancer; having a breast cancer diagnosis between stages I and III; being at least 18 years old; possessing fluency in both written and spoken Portuguese; and lacking any other physical or mental problems that could compromise one’s participation in this study. Among the 212 participants that were contacted, 205 agreed to participate in this study. The study participants’ mean age was 54.32 years (*SD* = 10.05, range = 29–82) and most participants were married (*n* = 143, 69.8%). The largest educational groups consisted of participants who had completed primary school (*n* = 59, 28.8%) or had some high school (*n* = 51, 24.9%). A plurality of sample participants were diagnosed with stage II breast cancer (*n* = 68, 33.2%), and the mean time since their diagnosis was 18.14 months (*SD* = 24.28). Other sociodemographic and clinical information can be found in Table 1.

**Table 1:** Demographics and clinical characteristics (*n* = 205).

Variable	Breast cancer sample (*n* = 205)	
	
	*N*	%
Age, years (*M, SD*)	54.32	10.05
Education		
Primary school	59	28.8
Some high school	51	24.9
High school graduate	36	17.6
Undergraduate degree	45	22.0
Graduate degree	14	6.8
Employment status		
Full-time	100	48.8
Unemployed	46	22.4
Retired	46	22.4
Housewife	13	6.3
Marital Status		
Married/ partnered	143	69.8
Divorced/ separated	25	12.1
Widowed	17	8.3
Single	20	9.8
Family Income		
Under 10.000€	86	42.0
10.000€ – 20.000€	59	28.8
20.001€ – 37.500€	33	16.1
37.501€ – 70.000€	11	5.4
Over 70.000€	7	3.4
Not reported	9	4.4
Time since diagnosis (*M, SD)*	18.14	24.28
Phase of treatment		
On chemotherapy	38	18.5
On radiotherapy	19	9.3
On hormonal therapy	120	58.5
On biological therapy	20	9.8
On clinical surveillance	19	9.3
Breast cancer stage		
1	40	19.5
2	68	33.2
3	31	15.1
No data	61	29.8
Surgical procedure		
Lumpectomy	104	50.7
Mastectomy	93	45.4
Adjuvant therapy		
Chemotherapy	165	80.5
Radiotherapy	137	66.8
Hormonal therapy	145	70.7
Biological therapy	45	22.0


*M = Mean. *SD* = Standard Deviation.*

### Procedures

This multi-center study was conducted between May 2012 and 2015 in three public hospitals, a private-practice clinic, and one breast cancer patients’ association with all of these institutions being located either in Oporto or Lisbon, Portugal. Ethical approval was obtained from the ethics committees of each of the medical institutions and the patients’ association, and the entire study was developed in accordance with the data protection guidelines stipulated by the Portuguese Data Protection Authority (CNPD; 8204/2012). Participation was voluntary and all participants were recruited by phone, before being invited for an interview with the main researcher. During this interview, the objectives and procedures of the study were explained and participants were asked to sign an informed consent form. Participants then completed a self-administered questionnaire concerning sociodemographic, clinical, and psychosocial variables. Furthermore, this study was part of a larger, longitudinal research project (Ramos et al., 2018).

### Measures

#### Sociodemographic and Clinical Information

Participants provided demographic information (age, marital/partner status, educational level, employment status, and family income), in addition to clinical information (time since diagnosis, breast cancer stage, most recent treatment phase, treatments undergone).

#### Posttraumatic Stress Disorder (PTSD)

Posttraumatic Stress Disorder was measured using the PTSD Checklist-Civilian Version (PCL-C; Weathers et al., 1993; Portuguese version: Melo et al., 2006). This measure includes 17 items assessed using a 5-point Likert scale ranging from 1 (*not at all*) to 5 (*extremely*), in addition to encompassing symptom severity scores pertaining to the three PTSD symptom group criteria: criterion B – reexperiencing (re-experiencing symptoms); criterion C – avoidance and numbing (avoidance and numbing symptoms); and criterion D – hyperarousal (hyperarousal symptoms), in accordance with the Diagnostic and Statistical Manual of Mental Disorders-IV criteria for PTSD (American Psychiatric Association [APA], 2013). Higher scores on the total scale of the PCL-C, which ranges from 17 to 85, indicate higher levels of PTSD. The PCL-C has demonstrated good reliability and validity (Weathers et al., 1993). Concerning the reliability and validity of the Portuguese version of the PCL-C among a sample of breast cancer patients, the Cronbach’s alphas ranged from 0.86 to 0.91 (Melo et al., 2006). In our sample, the PCL-C demonstrated excellent reliability (α = 0.93) for the total scale and good reliability for the sub-scale Reexperiencing (α = 0.84); Avoidance and Numbing (α = 0.80); Hyperarousal (α = 0.88).

#### Posttraumatic Growth (PTG)

Posttraumatic Growth experienced in the aftermath of a breast cancer diagnosis and breast cancer treatment was assessed using the Posttraumatic Growth Inventory (PTGI; Tedeschi and Calhoun, 1996; Portuguese version: Silva et al., 2009). The PTGI measures the perceived degree of positive life changes following a traumatic event, and it consists of five domains: Relating to Others, New Possibilities, Personal Strength, Spiritual Change, and Appreciation of Life. In Portuguese version (Silva et al., 2009), PTGI is comprised by four subscales, however, we will use the five sub-scales in accordance with original article and with Ramos and her colleagues (Ramos et al., 2016b). The 21 items are rated on a 6-point Likert scale, ranging from 0 (*I did not experience this change as a result of having breast cancer*) to 5 (*I experienced this change to a very great degree as a result of having breast cancer*). The scores range from 0 to 105, and higher scores indicate that a person perceived the development of greater PTG resulting from their traumatic experience. The PTGI has shown excellent internal consistency for the total scale (α = 0.90), as well as for the subscales (Relating to Others: α = 0.85; New Possibilities: α = 0.84; Personal Strength: α = 0.72; Spiritual Change: α = 0.85; Appreciation of Life: α = 0.67) (Tedeschi and Calhoun, 1996). The PTGI has also shown good psychometric properties in breast cancer Portuguese samples (Silva et al., 2009; Ramos et al., 2016b). In this study, the PTGI showed excellent reliability for the total score (α = 0.92) and good reliability for the subscales, with the Cronbach’s alphas for the subscales Relating to Others (α = 0.86); New Possibilities (α = 0.83); Personal Strength (α = 0.75); Spiritual Change (α = 0.73); and Appreciation of Life (α = 0.69).

#### Challenge to Core Beliefs

The Core Beliefs Inventory (CBI; Cann et al., 2010; Portuguese version: Ramos et al., 2016a) was used to measure the degree to which an individual examined their core beliefs about their personal strengths and weaknesses, human nature, relationships, the meaning of life, and religious and spiritual matters (Cann et al., 2010). The CBI includes 9 items (e.g., “I seriously examined the degree to which I believe the things that happen to people are fair”), rated on a 6-point Likert scale ranging from 0 (*not at all*) to 5 (*to a very great degree*). The scores range from 0 to 45, with higher scores indicating a greater tendency to challenge one’s core beliefs. The CBI has demonstrated good reliability and validity (Cann et al., 2010). The Portuguese version of the CBI reported a Cronbach’s alpha of 0.85 for the total scale (Ramos et al., 2016a). In our sample, the CBI demonstrated good reliability (α = 0.88).

#### Rumination

The Event Related Rumination Inventory (ERRI; Cann et al., 2011; Portuguese version: Ramos et al., 2015) was used to assess rumination associated with a traumatic event through two subscales – intrusive rumination (e.g., “Thoughts about the event that came to mind, and I could not stop thinking about them”) and deliberate rumination (e.g., “I deliberately thought about how the event had affected me.”). Each subscale is comprised of 10 items, in addition to using a 4-point Likert response format, ranging from 0 (*not at all*) to 3 (*often*). The participants answered according to the instructions presented in both subscales, which asked participants to “Indicate, for the following items, how often, if at all, you went through the experiences described during the last 2 weeks.” Higher scores indicate higher levels of intrusive thinking and deliberate thinking. The ERRI has demonstrated good reliability and validity (Cann et al., 2011). In the Portuguese version, the ERRI exhibited excellent internal consistency for the total scale (α = 0.94), as well as for the subscales (Intrusive Rumination: α = 0.95; Deliberate Rumination: α = 0.90) (Ramos et al., 2015). The ERRI also demonstrated excellent internal consistency in our sample for Intrusive Rumination (α = 0.96) and for Deliberate Rumination (α = 0.88).

#### Statistical Analysis

First, descriptive statistics (means, standard deviations, skewness, and kurtosis, and Cronbach’s alphas) were calculated for the demographic and psychosocial variables. In order to explore the presence and/or absence, in addition to the direction of the associations between PTSD and PTG, bivariate correlations were calculated using a Pearson’s correlation coefficient. Two multivariate multiple regression analyses were performed to examine the extent to which one’s core beliefs, intrusive rumination, and deliberate rumination predicted PTG and PTSD, separately. This test was selected in order to prevent a type 1 error that could arise due to the presence of multiple error rates in repeated regression analyses. A series of 21 univariate multiple regression analyses was computed using each PTGI items as the dependent variable, and challenge to core beliefs and deliberate rumination as the independent variables. Another item-level analysis, using challenge to core beliefs and deliberate rumination as predictors, was conducted for each the 17 items of the PCL-Checklist. Durbin-Watson statistics were calculated to detect the presence of autocorrelations among the residuals, using scores of approximately 2. We also checked for multicollinearity, utilizing a variance inflation factor (VIF) value of 5 (Hair et al., 2006). All statistical tests were two-tailed, and a *p*-value < 0.05 was considered significant. Statistical analyses were conducted using SPSS Version 24.0 (IBM Inc., Chicago, IL, United States).

## Results

### Descriptive Statistics and Correlations Among Variables

The descriptive statistics and Cronbach’s alphas are displayed in Table 2. Overall, participants reported low levels of PTSD (*M* = 2.27, *SD* = 0.97) and moderate levels of PTG (*M* = 3.09, *SD* = 1.09), as a result of their personal experiences dealing with breast cancer. PTG and PTSD presented a weak, but significant association (*r* = 0.17, *p* = 0.016).

**Table 2:** Descriptive statistics of PTG, PTSD, core beliefs, intrusive, and deliberate rumination (*n* = 205).

Variable	α	*M*	*SD*
(1) PTG	0.92	3.09	1.09
(2) PTSD	0.93	2.27	0.97
(3) Challenge to core beliefs	0.88	3.14	1.20
(4) Intrusive rumination	0.96	1.75	0.89
(5) Deliberate rumination	0.88	1.56	0.71


*α = Cronbach’s alpha. *M* = Mean. *SD* = Standard Deviation. PTG = Posttraumatic Growth. PTSD = Posttraumatic Stress Disorder.*

### Multiple Regression Analyses for PTGI and PCL-C

A multiple regression analysis utilizing the PTGI (total score) as the dependent variable and challenge to core beliefs, intrusive rumination, and deliberate rumination, as predictors, explained 31% [*F*(3,201) = 31.73, *p* < 0.001] of the variance in PTG. Both challenge to core beliefs (β = 0.46, *p* < 0.001) and deliberate rumination (β = 0.22, *p* = 0.011) significantly predicted PTG overall, with the former being the strongest predictor. Intrusive rumination was not significantly associated with PTG (β = -0.11, *p* = 0.172). The second multiple regression analysis explains 54% of the variance of the PCL-C total score [*F*(3,201) = 54.09, *p* < 0.001], with challenge to core beliefs (β = 0.20, *p* = 0.004) and intrusive rumination (β = 0.42, *p* < 0.001) being significant predictors of overall PTSD. Deliberate rumination was not significantly associated with PTSD symptoms (β = 0.14, *p* = 0.069). In accordance with multiple regressions’ results and with PTG’ model, the following item-level analyses were performed.

### Item-Level Analyses for PTGI Items and PCL-C Items

The results of the 21 follow-up univariate multiple regression analyses conducted to explain the PTGI items are displayed in Table 3. The item-level analysis of the PTGI showed that all 21 models were significant and that each model explained between 4% (item 14) and 22% (item 15) of the variance of the PTGI items. The challenge to core beliefs was a significant predictor for 17 of the 21 items, while deliberate rumination significantly predicted only 4 items, specifically, items 1, 3, 5, and 15. Two items – items 5 and 15 – were predicted by both variables.

**Table 3:** Multiple regression analyses using the core beliefs, and deliberate rumination predicting PTGI items (*n* = 205).

Item PTGI^a^	Core Beliefs^b^					Deliberate Rumination^c^							
					
	β	B	SE B	95%	CI (B)	β	B	SE B	95%	CI (B)	*R*^2^	*F*(2,202)	*p*
(1) I changed my priorities about what is important in life.	0.06	0.10	0.13	-0.16	0.36	**0.35^∗∗∗^**	0.90	0.22	0.47	1.34	0.14	18.12	<0.001
(2) I have a greater appreciation for the value of my own life.	0.25^∗∗^	0.34	0.12	0.11	0.57	0.11	0.24	0.20	-0.15	0.63	0.10	12.31	<0.001
(3) I developed new interests.	0.06	0.10	0.14	-0.18	0.38	**0.30^∗^**	0.83	0.24	0.37	1.30	0.11	13.74	<0.001
(4) I have a greater feeling of self-reliance.	**0.25^∗∗^**	0.35	0.12	0.11	0.60	0.10	0.23	0.21	-0.18	0.64	0.09	11.42	<0.001
(5) I have a better understanding of spiritual matters.	**0.34^∗∗∗^**	0.53	0.13	0.27	0.78	**0.16^∗^**	0.43	0.22	0.01	0.85	0.20	27.06	<0.001
(6) I more clearly see that I can count on people in times of trouble.	**0.37^∗∗∗^**	0.42	0.10	0.22	0.62	-0.09	-0.17	0.17	-0.51	0.16	0.09	11.21	<0.001
(7) I established a new path for my life.	0.16	0.27	0.15	-0.02	0.56	0.15	0.42	0.25	-0.06	0.91	0.07	9.02	<0.001
(8) I have a greater sense of closeness with others.	**0.30^∗∗^**	0.46	0.13	0.20	0.72	0.06	0.14	0.22	-0.29	0.58	0.11	13.37	<0.001
(9) I am more willing to express my emotions.	**0.38^∗∗∗^**	0.56	0.13	0.31	0.81	0.03	0.09	0.21	-0.33	0.51	0.15	18.98	<0.001
(10) I know better that I can handle difficulties.	**0.28^∗∗^**	0.35	0.11	0.13	0.58	-0.00	-0.00	0.19	-0.37	0.37	0.07	8.54	<0.001
(11) I am able to do better things with my life.	**0.20^∗^**	0.30	0.13	0.04	0.57	0.09	0.23	0.23	-0.21	0.68	0.06	7.82	0.001
(12) I am better able to accept the way things work out.	**0.27^∗∗^**	0.33	0.11	0.11	0.55	-0.02	-0.04	0.19	-0.41	0.32	0.06	7.01	0.001
(13) I can better appreciate each day.	**0.29^∗∗^**	0.40	0.12	0.16	0.63	0.07	0.15	0.20	-0.25	0.55	0.10	12.48	<0.001
(14) New opportunities are available which wouldn’t have been otherwise.	0.13	0.19	0.14	-0.08	0.46	0.13	0.33	0.23	-0.12	0.79	0.04	5.69	0.004
(15) I have more compassion for others.	**0.33^∗∗∗^**	0.53	0.13	0.27	0.79	**0.19^∗^**	0.52	0.22	0.08	0.96	0.22	29.31	<0.001
(16) I put more effort into my relationships.	**0.29^∗∗^**	0.44	0.13	0.18	0.70	0.12	0.31	0.22	-0.13	0.75	0.13	16.47	<0.001
(17) I am more likely to try to change things which need changing.	**0.29^∗∗^**	0.42	0.12	0.18	0.67	0.11	0.27	0.21	-0.14	0.68	0.13	16.26	<0.001
(18) I have a stronger religious faith.	**0.34^∗∗∗^**	0.59	0.14	0.31	0.87	0.15	0.43	0.24	-0.04	0.90	0.20	25.84	<0.001
(19) I discovered that I’m stronger than I thought I was.	**0.37^∗∗∗^**	0.50	0.12	0.27	0.73	-0.02	-0.04	0.19	-0.42	0.34	0.12	15.16	<0.001
(20) I learned a great deal about how wonderful people are.	**0.40^∗∗∗^**	0.53	0.11	0.31	0.74	0.04	0.08	0.19	-0.29	0.45	0.17	21.67	<0.001
(21) I better accept needing others.	**0.46^∗∗∗^**	0.66	0.12	0.43	0.89	-0.02	-0.06	0.20	-0.45	0.34	0.19	24.78	<0.001


*^*a*^PTGI – Posttraumatic Growth Inventory (Tedeschi and Calhoun, 1996). ^*b*^Core Beliefs Inventory. ^*c*^Event Related Rumination Inventory. The bold values mean that the standardized beta is statistically significant for a two-tailed test and a *p*-value < 0.05. ^∗^*p* < 0.05; ^∗∗^*p* < 0.01; and ^∗∗∗^*p* < 0.001.*

As shown in Table 4, the results of the PCL-C item-level analysis demonstrated the significance of 16 models. The model for Item 8, which concerned “Trouble remembering important parts of a stressful experience from the past?,” was not significantly explained by any of the variables. Challenges to core beliefs and intrusive rumination explained between 9% and 40% of the variance for each of the 16 PCL-C items. Intrusive Rumination was a significant predictor of 16 items while challenge to core beliefs significantly predicted 12 items. Twelve items were significantly explained by both variables, including items 1,2,3,4,5,6,7,13,14,15,16, and 17.

**Table 4:** Multiple regression analyses using core beliefs, and intrusive rumination predicting PCL-C items (*n* = 205).

Item PCL-C^a^	Core beliefs^b^					Intrusive Rumination^c^							
					
	β	B	SE B	95%	CI (B)	β	B	SE B	95%	CI (B)	*R*^2^	*F*(2,202)	*P*
(1) Repeated, disturbing memories, thoughts, or images of a stressful experience from the past?	0.20^∗∗^	0.24	0.08	0.08	0.39	**0.51^∗∗∗^**	0.84	0.10	0.63	1.04	0.40	69.69	<0.001
(2) Repeated, disturbing dreams of a stressful experience from the past?	**0.18^∗^**	0.19	0.08	0.03	0.36	**0.32^∗∗∗^**	0.48	0.11	0.36	0.70	0.19	24.42	<0.001
(3) Suddenly acting or feeling as if a stressful experience were happening again (as if you were reliving it)?	**0.21^∗∗^**	0.24	0.08	0.09	0.39	**0.46^∗∗∗^**	0.71	0.10	0.51	0.92	0.35	55.41	<0.001
(4) Feeling very upset when something reminded you of a stressful experience from the past?	**0.29^∗∗∗^**	0.37	0.09	0.19	0.54	**0.32^∗∗∗^**	0.54	0.12	0.31	0.78	0.28	40.18	<0.001
(5) Having physical reactions (e.g., heart pounding, trouble breathing, or sweating) when something reminded you of a stressful experience from the past?	**0.17^∗^**	0.22	0.10	0.02	0.41	**0.18^∗^**	0.31	0.14	0.04	0.57	0.09	10.47	<0.001
(6) Avoid thinking about or talking about a stressful experience from the past or avoid having feelings related to it?	**0.15^∗^**	0.19	0.09	0.01	0.37	**0.37^∗∗∗^**	0.61	0.12	0.37.	85	0.21	27.87	<0.001
(7) Avoid activities or situations because they remind you of a stressful experience from the past?	**0.18^∗^**	0.21	0.09	0.03	0.38	**0.32^∗∗∗^**	0.51	0.12	0.28	0.75	0.19	24.18	<0.001
(8) Trouble remembering important parts of a stressful experience from the past?	0.07	0.05	0.07	-0.08	0.19	0.12	0.13	0.09	-0.05	0.31	0.02	2.81	0.063
(9) Loss of interest in things that you used to enjoy?	0.12	0.14	0.09	-0.03	0.31	**0.34^∗∗∗^**	0.52	0.12	0.30	0.75	0.17	21.17	<0.001
(10) Feeling distant or cut off from other people?	0.13	0.13	0.08	-0.03	0.30	**0.24^∗∗^**	0.34	0.11	0.12	0.57	0.10	12.02	<0.001
(11) Feeling emotionally numb or being unable to have loving feelings for those close to you?	0.11	0.11	0.08	-0.05	0.26	**0.26^∗∗^**	0.35	0.11	0.14	0.56	0.10	12.30	<0.001
(12) Feeling as if your future will somehow be cut short?	0.09	0.11	0.09	-0.08	0.29	**0.35^∗∗∗^**	0.59	0.13	0.34	0.84	0.16	20.11	<0.001
(13) Trouble falling or staying asleep?	**0.24^∗∗^**	0.32	0.10	0.13	0.51	**0.33^∗∗∗^**	0.59	0.13	0.33	0.85	0.24	32.94	<0.001
(14) Feeling irritable or having angry outbursts?	**0.26^∗∗^**	0.34	0.10	0.15	0.53	**0.35^∗∗^**	0.43	0.13	0.18	0.69	0.19	25.10	<0.001
(15) Having difficulty concentrating?	**0.19^∗∗^**	0.24	0.09	0.06	0.42	**0.40^∗∗∗^**	0.70	0.12	0.46	0.95	0.27	38.76	<0.001
(16) Being “super alert” or watchful on guard?	**0.24^∗∗^**	0.31	0.09	0.13	0.48	**0.35^∗∗∗^**	0.60	0.12	0.36	0.84	0.27	38.07	<0.001
(17) Feeling jumpy or easily startled?	**0.18^∗^**	0.23	0.09	0.05	0.41	**0.39^∗∗∗^**	0.67	0.12	0.43	0.91	0.25	35.54	<0.001


*^*a*^PCL-C – Posttraumatic Stress Disorder Checklist (Weathers et al., 1993). ^*b*^Core Beliefs Inventory. ^*c*^Event Related Rumination Inventory. The bold values mean that the standardized beta is statistically significant for a two-tailed test and a *p*-value <0.05. ^∗^*p* < 0.05; ^∗∗^*p* < 0.01; and ^∗∗∗^*p* < 0.001.*

## Discussion

The main objective of the current study was to investigate within-scale differences in the PTGI and in the PCL-C by testing the roles of challenge to core beliefs, intrusive rumination, and deliberate rumination.

Regarding the PTGI, only 2 items were predicted by both challenge to core beliefs and deliberate rumination, namely, item 5 – “*I have a better understanding of spiritual matters*” and item 15 – “*I have more compassion for others*.” These two items correspond to the Spiritual Change and Relating to Others sub-scales of the PTGI, respectively, and are more likely to change if the experience of having breast cancer leads a woman to examine her existing beliefs and deliberate thinking about these issues in the context of her personal experiences, in line with the PTG model. However, most items in the PTGI are significantly accounted for challenge to core beliefs, since this variable predicts 17 of the 21 items of the PTGI. A previous item-level examination of the PTGI further showed that PTG is more likely to occur when core beliefs are challenged (Taku and Oshio, 2015). However, in that same study, which examined a sample of young adults who experienced the Great East Japan Earthquake, all of the 21 PTGI items were explained by an examination of core beliefs. The severity of the event could explain the difference between Taku et al’s. (2015) study and our research. Moreover, Taku et al. (2015) found that the severity of the event had a significant effect on a challenge to core beliefs. The authors further argued that core beliefs re-examination was not likely to occur solely as a result of highly challenging life circumstances, since they found no differences between the moderate and high exposure groups to this traumatic event (Taku et al., 2015).

This confirmation of the comprehensive predictive value of this variable (i.e., Challenge to core beliefs) on PTG is consistent with previous studies regarding patients diagnosed with leukemia (Danhauer et al., 2013) and prostate cancer (Wilson et al., 2014), in addition to studies examining samples that have experienced other traumatic events (Cann et al., 2010; Triplett et al., 2012; Lindstrom et al., 2013; Taku et al., 2015; Zhou et al., 2015).

According to the theoretical model of PTG (Tedeschi and Calhoun, 1996, 2004), it is to be expected that, after the disruption of one’s core beliefs when confronting breast cancer, women will begin to engage in constructive, positive, and deliberate thinking in an attempt to attribute meaning to this traumatic experience. Previous studies have demonstrated that deliberate rumination is positively associated with the development of PTG and that is a strong predictor of PTG (Morris and Shakespeare-Finch, 2011; Triplett et al., 2012; Lindstrom et al., 2013; Dong et al., 2015; Zhou et al., 2015). In fact, deliberate rumination, and not intrusive rumination, is the variable that directly causes PTG (Stockton et al., 2011). Our study results demonstrated that, although deliberate rumination was significantly associated with PTG, only 4 of the 21 PTGI items were explained by deliberate rumination, in contrast to Taku and Oshio (2015) item-level analysis, which found that deliberate rumination explained 12 of the 21 PTGI items. This difference could be due to disparities in the amount of time that had passed since the traumatic event, given that the participants of this study completed the study questionnaire a shorter amount of time following their breast cancer diagnosis (18 months after their diagnosis, on average) when compared to participants in the Taku and Oshio (2015) study, who replied to the study questionnaire an average of 27 months after experiencing the traumatic event. In accordance with the theoretical model of PTG (Calhoun and Tedeschi, 2004), deliberate rumination is more likely to occur at a later point in time following a traumatic event, rather than in the immediate aftermath of an event, as it tends to occur as a consequence of an examination of core beliefs. Moreover, deliberate rumination, unlike challenge to core beliefs, exclusively explains item 1 – “*I changed my priorities about what is important in life*” and item 3, “*I developed new interests*,” which represent a path to positive changes that may not require the examination of one’s challenge to core beliefs. Moreover, our findings demonstrated that deliberate rumination predicts a total of four items from each subscale, with the exception of the Personal Strength subscale. The variance of this domain is only explained by the challenge to core beliefs, entailing the examination of an individual’s life-orienting principles, which triggers the perception of possessing greater individual strength to solve future challenges and overcome adversity.

Globally, the challenge to core beliefs explained the higher amount of PTG variance among women with a breast cancer diagnosis. Similarly, Danhauer et al. (2013) demonstrated that an examination of one’s core beliefs was not only a major predictor of PTG, but was also more strongly associated with PTG than deliberate rumination.

Regarding the PCL-C item-level analysis, with the exception of item 8, which was not significant, intrusive rumination predicts all PCL-C items, suggesting that PTSD symptoms are not likely develop without intrusive thinking. The variance of two items from the Avoidance and Numbing subscale were explained by both challenge to core beliefs and intrusive rumination, namely, item 6 – “*Avoid thinking about or talking about a stressful experience from the past or avoid having feelings related to it?*” and item 7 – “*Avoid activities or situations because they remind you of a stressful experience from the past?*.” Intrusive rumination fully explains the remaining four items of Avoidance and Numbing criteria (e.g., “*Loss of interest in things that you used to enjoy?*”), which signifies that negative, automatic, and intrusive ways of thinking about a traumatic experience are often the basis for the avoidance of trauma-related stimuli (e.g., thoughts, feelings, or reminders), after experiencing a traumatic event. The examination of core beliefs predicts 12 of the 17 PCL-C items, which are represented by the Reexperiencing and Avoidance and Numbing dimensions, with items from both dimensions being significantly predicted by both an examination of core beliefs and by intrusive rumination. Other studies have confirmed that an examination of one’s core beliefs is positively linked to trauma-related perceived stress, such as PTSD symptoms (Zhou et al., 2015) or distress (Triplett et al., 2012; Wilson et al., 2014).

In summary, our findings demonstrated that challenges to one’s core beliefs can lead to both PTG and PTSD, and that intrusive rumination only predicts PTSD and deliberate rumination only predicts PTG, which is in accordance with the previous findings of Zhou et al. (2015). Through the use of Structural Equation Modeling (SEM), Zhou et al. (2015) we found that challenges to one’s core beliefs predicted both PTSD and PTG, while intrusive rumination only predicted PTSD, and deliberate rumination solely predicted PTG, as they describe in the following excerpt, “*there were certain predictive factors that have an initial effect on both PTSD and PTG, but these two outcomes later diverge, progressing to manifest via different developmental processes*” (Zhou et al., 2015, p. 295). Thus, although core beliefs’ examination played an important and primary role in the development of both constructs, the distinct contribution of each type of rumination to both PTSD and PTG seems to suggest the presence of a distinct underlying cognitive mechanism in their developmental pathways, since the variables (i.e., PTSD and PTG) are being viewed as two independent and separate dimensions of the struggle with a traumatic experience (Linley et al., 2008; Zhou et al., 2018).

In fact, taking into account the results of our research, we find that PTSD and PTG were significantly associated but they have distinct developmental processes, also in women with breast cancer. Another study using a sample of women with breast cancer (Chan et al., 2011) also suggested that PTSD and PTG have distinct pathways, as its main findings demonstrated that cancer-related negative rumination is more closely related to PTSD symptoms, and that cancer-related positive rumination is positively associated with PTG, but not with PTSD. Our findings build upon these perspectives, while increasing the knowledge base regarding the processes of PTG and PTSD. Although both PTG and PTSD were significantly associated in this study sample, the results from the item-level analyses also allowed this research to understand which items from both constructs were explained by the core beliefs challenge, and which items were explained by each type of rumination, among a sample of women who had been diagnosed and treated for breast cancer.

### Limitations

There are several limitations to this study. First, the small sample size and the sociodemographic characteristics of the sample, which could be described as being primarily middle-aged, middle-class, and possessing low levels of educational attainment, may limit the generalization of its findings. A second limitation concerns this study’s cross-sectional design, given that causality cannot be determined from this type of study design. Further research is needed to examine the processes of PTG and PTSD, longitudinally, in order to obtain corroborating evidence concerning the impact of several factors on the developmental processes of both variables. The third limitation of this research regards the use of self-reported measurements to evaluate the presence of PTSD symptoms. The use of clinical interviews as a complementary form of assessment, in conjunction with the use of the PCL-5 scale (instead of PCL-C), according to the DSM-V criteria, is suggested for future studies, in order to establish a more accurate diagnosis of the presence of PTSD symptoms. Fourth, this study was conducted using a sample composed of women with non-metastatic breast cancer, and; therefore, generalizations of its findings to people with other types of cancer and/or who have experienced other kinds of traumatic events must be made with caution. Further research utilizing non-clinical, larger and more comprehensive samples is recommended.

In spite of these limitations, this research provides a well-founded and novel contribution to the field of PTG and PTSD studies, and is the first of its kind to examine the within-scale differences of PTG and PTSD by examining the effects of challenge to core beliefs and rumination (intrusive and deliberate) in each of the scale items. Our results clarified, at an item-level analysis, that the challenge to core beliefs and intrusive rumination are the main predictors of PTG and PTSD symptoms, respectively; and that the challenge to core beliefs also plays an important role in the development of PTSD, which allow to increase the level of knowledge about the genesis of these two variables, particularly, women with breast cancer. Our contribution to a comprehensive knowledge about PTG and PTSD development processes may potentiate a more accurate psychological and medical support to women with breast cancer diagnosis.

## Ethics Statement

All subjects gave written informed consent in accordance with the Declaration of Helsinki.

## Author Contributions

CR designed the study, analyzed and intepreted the data, and wrote and edited the manuscript. IL and RGT designed the study and wrote and edited the manuscript. PAC analzyed the statistics and wrote and edited the manuscript. ART designed the study and collected and edited the data.

## Conflict of Interest Statement

The authors declare that the research was conducted in the absence of any commercial or financial relationships that could be construed as a potential conflict of interest.
